# Daily steps are low year-round and dip lower in fall/winter: findings from a longitudinal diabetes cohort

**DOI:** 10.1186/1475-2840-9-81

**Published:** 2010-11-30

**Authors:** Kaberi Dasgupta, Lawrence Joseph, Louise Pilote, Ian Strachan, Ron J Sigal, Cathy Chan

**Affiliations:** 1 Department of Medicine, Division of Clinical Epidemiology, McGill University Health Centre, 687 Pine Avenue West, Montreal, Canada; 2 Department of Natural Resource Sciences, McGill University, 21111 Lakeshore Road, Ste Anne de Bellevue, Canada; 3 Department of Medicine, University of Calgary, 3330 Hospital Drive NW, Calgary, Canada; 4 Department of Agricultural, Food & Nutritional Sciences, University of Alberta, 7-55 Medical Sciences Building, Edmonton, Canada

## Abstract

**Background:**

Higher walking levels lead to lower mortality in type 2 diabetes, but inclement weather may reduce walking. In this patient population, we conducted a longitudinal cohort study to objectively quantify seasonal variations both in walking and in two vascular risk factors associated with activity levels, hemoglobin A1C and blood pressure.

**Methods:**

Between June 2006 and July 2009, volunteer type 2 diabetes patients in Montreal, Quebec, Canada underwent two weeks of pedometer measurement up to four times over a one year follow-up period (i.e. once/season). Pedometer viewing windows were concealed (snap-on cover and tamper proof seal). A1C, blood pressure, and anthropometric parameters were also assessed. Given similarities in measures for spring/summer and fall/winter, and because not all participants completed four assessments, spring and summer values were collapsed as were fall and winter values. Mean within-individual differences (95% confidence intervals) were computed for daily steps, A1C, and systolic and diastolic blood pressure, by subtracting spring/summer values from fall/winter values.

**Results:**

Among 201 participants, 166 (82.6%) underwent at least one fall/winter and one spring/summer evaluation. Approximately half were women, the mean age was 62.4 years (SD 10.8), and the mean BMI was 30.1 kg/m^2 ^(SD 5.7). Step counts averaged at a sedentary level in fall/winter (mean 4,901 steps/day, SD 2,464) and at a low active level in spring/summer (mean 5,659 steps/day, SD 2,611). There was a -758 (95% CI: -1,037 to -479) mean fall/winter to spring/summer within-individual difference. There were no significant differences in A1C or in anthropometric parameters. Systolic blood pressure was higher in fall/winter (mean 137 mm Hg, SD 16) than spring/summer (133 mm Hg, SD 14) with a mean difference of 4.0 mm Hg (95% CI: 2.3 to 5.7).

**Conclusions:**

Daily step counts in type 2 diabetes patients are low, dipping lower during fall/winter. In this medication-treated cohort, A1C was stable year-round but a fall/winter systolic blood pressure increase was detected. Our findings signal a need to develop strategies to help patients increase step counts year-round and prevent both reductions in step counts and increases in blood pressure during the fall and winter.

## Background

Diabetes patients have a two to four-fold increased risk for cardiovascular disease compared to the general population [1-3]. Even among those with established diabetes higher activity levels predict lower vascular disease rates and improved survival; walking has been specifically proven to confer benefits. For example, in the National Health Interview Survey, adults with diabetes who reported walking more than two hours per week had a 34% reduction in vascular events up to 14 years later compared to those walking less [4]. Similarly, in the Nurses' Health Study, women with diabetes in the highest quartile for self-reported walking were 34% less likely to have died up to 8 years later compared to women in the lowest quartile [5]. Although more than 50% of diabetes patients report a preference for walking over other forms of physical activity [6], walking is underutilized by these patients [7]. It is therefore important to examine barriers to walking in diabetes patients, so as to develop more effective strategies to increase walking levels.

In temperate climates, the lower temperatures and precipitation characteristic of fall and winter may impede walking. To examine the importance of season as a determinant of walking in diabetes patients, we conducted a longitudinal cohort study examining pedometer-measured "daily steps" over a one year follow-up period. In addition to assessing for seasonal variations in daily steps, we were interested in measuring any seasonal changes in glycemic control and in blood pressure, two vascular risk factors that may be impacted by physical activity levels. Studies from Japan [8,9], China [10], Sweden [11], the United Kingdom [12], and the United States [13] suggest that post winter A1C values may be 0.13% to 0.6% higher than summer values and, in nondiabetic populations, higher blood pressure has been reported during the fall/winter months [14-16]. No previous study has concurrently examined for seasonal differences in both physical activity levels and vascular risk factors.

## Methods

Our study was conducted in Montreal, Quebec, Canada, a city with a humid continental climate and abundant winter snowfall. Specifically, between 1971 and 2000, the mean temperature in Montreal by season was respectively:-8.3°C (SD 3.0°C) for winter, 5.6°C (SD 1.9°C) for spring, 19.6°C (SD 1.1°C) for summer, and 8.1°C (SD 1.5°C) for fall. Over this period, the mean cumulative precipitation (rain and snow) by season was respectively: 221.1 mm (SD 32.8 mm) for winter, 227.9 mm (SD 32.0 mm) for spring, 267.1 mm (SD 35.0 mm) for summer, and 263.0 mm (SD 39.7 mm) for fall.

Previously published [17], our study design and methods are summarized here. Our original goals were to estimate the post winter to summer change in glucose control (i.e. spring to fall A1C difference); determine whether this change is antedated by a winter to spring change in walking; and to determine whether the winter to spring change in walking differs between men and women. Although blood pressure assessments were not detailed in our published protocol [17], we opted to examine systolic and blood pressure seasonal variations as a secondary outcome, given previous studies in nondiabetic populations demonstrating such variations [14-16]. Further, given the similarity of data from spring and summer and from fall and winter, we have performed analyses combining data from spring and summer and from fall and winter, so as to maximize the precision of our estimates.

Procedures were approved by the Institutional Review Boards (IRB) of McGill University and participating institutions (McGill University Health Centre, Sir Mortimer Davis Jewish General Hospital, Centre de Santé et de Services Sociaux de la Montagne). Recruitment was conducted through clinics and local diabetes associations using posters, presentations, and clinic staff referrals. Candidates required a physician diagnosis of type 2 diabetes. To permit accurate pedometer-based measurement, a body mass index (BMI) less than 40 kg/m^2 ^and a normal gait [18] were necessary. If stable, the presence of other chronic conditions was not an exclusion criterion. After providing informed consent, participants were asked to present once per season to the study centre over a one-year period. Venous blood was sampled for A1C measurement (Bio-Rad Variant II system) and blood pressure (15-minute rest; left arm; Omron HEM 747 IC) and anthropometric parameters were directly assessed (weight, using SECA 882 electronic scale; height, using SECA 214 stadiometer; and waist and hip circumferences).

Yamax SW-701 pedometers were used to measure step counts. At all assessments, participants were provided with three pedometers as well as a padded, pre-addressed, pre-stamped envelope. The pedometer viewing windows were concealed with snap-on covers and tamper-proof seals to reduce the likelihood that participants would alter their behaviour in response to the step count value recorded. Participants were instructed to wear Pedometer A for one week, Pedometer B for a second week, and then mail back these pedometers along with Pedometer C, which served to capture the "postman steps" that occur during the mailing process. The step counts recorded on Pedometers A and B were corrected by subtracting those recorded on Pedometer C. Corrected values were then summed and divided by the total number of days the two pedometers had been worn (usually 14 days) for an estimate of steps/day. As per the classification scheme proposed by Tudor-Locke and Bassett, step counts may be used to categorize individuals as "high active" (> 12,500 steps/day), "active" (10,000 to 12, 499 steps/day), "somewhat active" (7,500 to 9,999 steps/day), "low active" (5,000 to 7,499 steps/day) or "sedentary" (< 5,000 steps/day) [19].

In addition to this direct assessment of daily steps, we also administered the short form of the International Physical Activity Questionnaire (last 7 days) which allowed for computation of self-reported total physical activity (metabolic equivalent-minutes/week) [20]. Participants were administered the Food Frequency Questionnaire (FFQ) developed by Shatenstein and colleagues and previously validated using four non-consecutive 1-day food records [21]; data from the FFQ provided estimates of both carbohydrate and salt intake, which may respectively impact glycemic and blood pressure control.

Assuming a maximum standard deviation (SD) of 5,000 steps/day, a sample of 80 individuals was deemed sufficient to estimate a between-season walking difference to within +/- 1,100 steps or better using a 95% confidence interval. Additionally, using a maximum SD of 0.94% for A1C values as suggested by a limited number of previous studies [9,12,13], a sample of 152 individuals was deemed adequate to permit an estimate of between-season A1C differences within +/- 0.15% using a 95% confidence interval. In order to capture both potential between-season differences in daily steps and A1C, and to allow for the possibility of dropouts in a longitudinal study, we aimed to recruit approximately 200 subjects.

Participant characteristics were summarized using means, standard deviations, medians, interquartile ranges, and proportions, as appropriate. Fall was defined as September-November; winter as December-February; spring as March-May; and summer as June-August. Mean values across individuals were plotted for daily steps, self-reported physical activity, anthropometric parameters, A1C, and blood pressure. With respect to the original study aims, the post winter to summer difference in glycemic control was computed by subtracting the fall A1C (i.e. reflective of summer glycemic control) from the spring A1C (i.e. reflective of winter glycemic control). The winter to spring difference in daily steps was computed by subtracting the spring daily steps from those in winter, for men and women separately and combined.

With respect to the analyses that involved combining fall with winter and spring with summer values, spring/summer values were subtracted from the fall/winter values (fall/winter-spring/summer difference) for each participant with respect to daily steps, A1C, systolic and diastolic blood pressure, body mass index, self-reported physical activity, and carbohydrate and salt intake. Additionally, for A1C, summer/fall values (i.e. reflective of spring/summer glycemic control and habits) were subtracted from winter/spring values (i.e. reflective of fall/winter glycemic control).

For each variable, differences were averaged and 95% CIs were computed. Mean differences were not further adjusted as these were mean *within-individual *differences and thus each participant served as his/her own control. We performed sensitivity analyses which included only those participants without changes in the number of antihypertensive or antihyperglycemic medications. Associations of step count differences with clinically important differences in other variables were examined through multivariate linear regression: keeping our primary variable (daily step count difference) in each model, we assessed confounding by comparing the beta-coefficient for daily step count difference as potential co-variates and confounders entered and exited the models.

## Results

**Figure 1:**
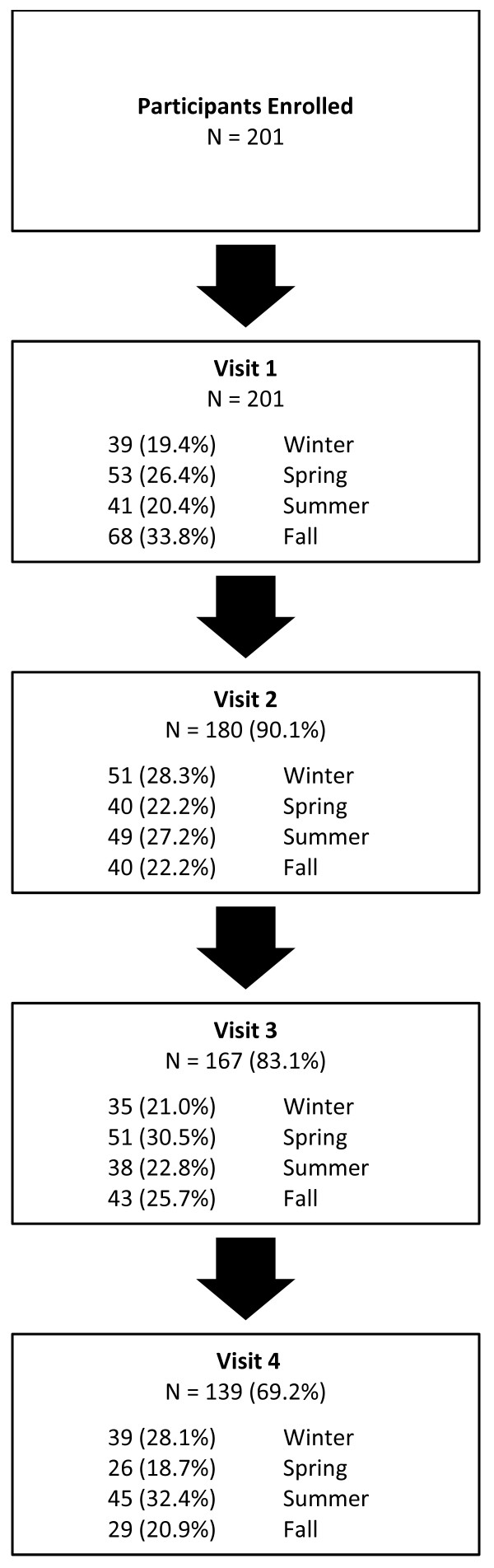
**Participant flow**.

A total of 201 participants were recruited between June 2006 and June 2008 and final evaluations were completed by July 2009. Participant retention remained above 80% by the third visit but fell to slightly under 70% by the fourth visit (Figure 1). Among a total of 687 visits, 164 occurred in winter (23.8%), 170 in spring (24.7%), 173 in summer (25.1%), and 180 in fall (26.2%). One hundred and sixty-six participants (82.6%) were evaluated at least once during both fall/winter and spring/summer periods. Among the remaining 35 individuals, reasons for not presenting for at least a second visit included lack of interest (46%), distance from study centre (3%), family illness (3%), moving/travel (9%), unstable co-morbid illness or diabetes other than type 2 (11%), or was unspecified. These participants did not differ importantly from the 166 retained in our fall/winter vs. spring/summer analyses (Table 1). For the years included in our study, all seasonal mean temperatures were within one standard deviation of usual seasonal mean temperatures (see Methods) but precipitation was above average levels for several seasonal periods (i.e. 316.2 mm for Fall 2006, 278.6 mm for spring 2007, 325.8 mm for winter 2008, and 304.9 mm for winter 2009).

**Table 1:** Baseline characteristics

	Total N = 201	Both Fall/Winter and Spring/Summer Data N = 166
Women, %	46.8	48.8
Age, years, mean (SD)	62.2 (10.5)	62.4 (10.8)
Diabetes, years, median (IQR)	8 (3 to 13)	8 (3 to 13)
Married/common-law,%	68.9^**a**^	68.6
White, %	69.2	69.3
Immigrant, %	46.3	44.6
Education, %		
High school	24.8	24.7
College	22.4	21.7
University	38.8	40.4
Depressed mood, %^b^	27.4	25.3
Current smoking, %	9.5^**a**^	9.7
Cardiovascular Disease %	16.9	18.7
Physical activity		
Walking, steps/day, mean (SD)	5,365 (2,655)	5,308 (2,477)
Self-reported total activity, metabolic equivalent-minutes/week, median (IQR)^c^	1,965^d ^(777 to 3,450)	1,965^e ^(849 to 3,417)
Anthropometric measures, mean (SD)		
BMI, kg/m^2^	30.4 (5.6)	30.1 (5.7)
Waist circumference, cm		
Women	99.2 (12.9)	98.6 (13.6)
Men	104.5 (12.9)	103.7 (13.0)
Waist/hip		
Women	0.88 (0.07)	0.88 (0.06)
Men	0.97 (0.06)	0.96 (0.06)
Intake, median (IQR)^f^		
Total energy, kcal/day	1,676 (1,770 to 2,089)	1,723 (1,256 to 2,171)
Carbohydrates, g/day	177 (125 to 232)	180 (126 to 243)
Salt, mg/day	2,348 (1,569 to 3,103)	2,414 (1,699 to 3,225)
A1C, mean (SD)	7.6 (1.4)	7.6 (1.4)
Antihyperglycemics, median (IQR)	2 (1 to 2)	2 (1 to 2)
Insulin, %	32.8	34.9
Blood Pressure, mm Hg, mean (SD)		
Systolic	138 (17)	137 (17)
Diastolic	80 (11)	81 (10)
Antihypertensives, median (IQR)	2 (1 to 3)	2 (1 to 3)
Statin, %	77.6	75.9

SD, standard deviation; IQR, interquartile range

^a ^N = 180 because assessed at second visit

^b ^Center for Epidemiological Studies-Depression Scale with score above 16 indicated depressed mood

^c ^International Physical Activity Questionnaire (Short Last 7 days format) [20]

^d ^N = 167 who provided sufficient data for computation

^e ^N = 137 who provided sufficient data for computation

^f ^Shatenstein and colleagues' Food Frequency Questionnaire [21].

**Figure 2:**
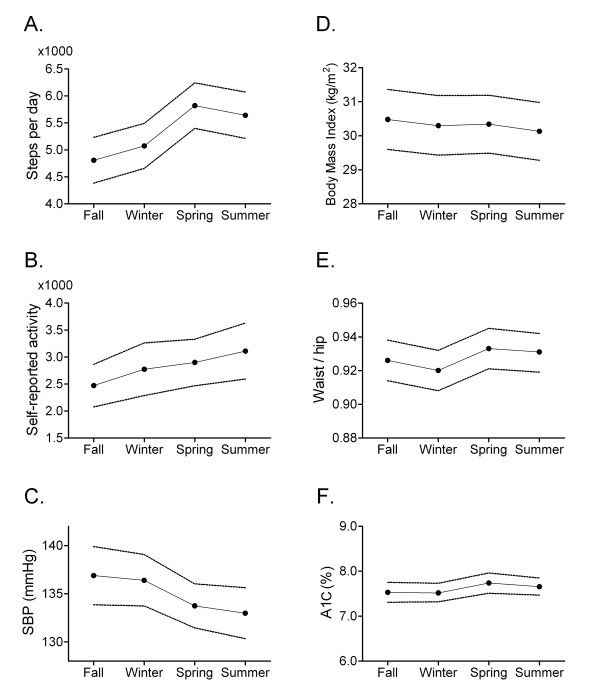
**Seasonal patterns in step counts (A), self-reported activity (B), blood pressure (C), anthropometric measures (D, E), and hemoglobin A1C (F)**. Mean values with 95% confidence intervals indicated by broken lines. SBP, systolic blood pressure; A1C, hemoglobin A1C.

Roughly equal numbers of women and men were recruited and participants were, on average, middle aged to elderly with an overweight to obese BMI. Fewer than 10% smoked cigarettes. Mean A1C and blood pressure values were slightly above usual treatment targets. Nearly one third used insulin and over three quarters used a statin. Over one quarter were non-White and over 40% were immigrants. Roughly 40% were university-educated. Less than one fifth had a past history of vascular disease. The Pearson correlation coefficient for pedometer A and B values was 0.70 with a 95% confidence interval of 0.65 to 0.73. Subtracting pedometer A from B values, the average difference was -166 steps/day (95% confidence interval: -347 to 14 steps/day), suggesting slightly higher step counts during the first week of each two-week pedometer measurement period. Daily step counts were in the sedentary to low active range, on average, as per the classification scheme proposed by Tudor-Locke and Bassett [19]. Plots of variables by season suggested lower step counts and self-reported physical activity in the fall and winter as well as higher systolic blood pressure levels (Figure 2).

With respect to the original study aims, the spring minus fall difference in A1C was 0.12% with a 95% confidence interval of -0.04% to 0.28%. The difference was thus in the anticipated direction (i.e. higher spring A1C than fall indicating higher glucose levels in winter than summer) but the confidence interval does not exclude an absence of difference. When analyses were restricted to participants without any change in antihyperglycemic medications, the spring minus fall differences in A1C was still 0.12% with a 95% CI of 0 to 0.25%. Participants walked an average of 777 steps/day more in the spring than in the winter (95% CI 408 to 1,145 steps/day). The daily step difference was similar in men and women. Men walked an average of 581 steps/day more in the spring than in the winter (95% CI 7 to 1,170 steps/day) while women walked an average of 984 steps/day more in the spring than in the winter (95% CI 540 to 1.428 step/day).

When seasons were combined to increase the precision of estimates, no clinically important differences in A1C or anthropometric parameters were detected (Table 2). Participants completed 758 fewer steps/day in the fall/winter compared to the spring/summer (95% CI 479 to 1,037 steps/day). Like step counts, self-reported physical activity was also lower in the fall/winter than spring/summer, with 450 MET-minutes/week fewer in the fall/winter (95% CI 30.4 to 872 MET-minutes/week). Additionally, systolic blood pressure was 4.0 mm Hg higher in the fall/winter period than in the spring/summer period (95% CI 2.3 to 5.7 mm Hg) and diastolic blood pressure was 1.4 mm Hg higher (95% CI 0.4 to 2.4 mm Hg). Salt intake may have been slightly higher in fall/winter than in spring/summer (51.9 mg/day, 95% CI -86.3 to 190 mg/day) but confidence intervals were wide. There were no clinically important differences in total energy intake or anthropometric parameters.

**Table 2:** Fall/Winter to Spring/Summer Differences

	Fall/Winter, mean (SD) across individuals	Spring/Summer, mean (SD) across individuals	Within-Individual Fall/Winter to Spring/Summer Differences (95% CI)
Objective measures			
Walking, steps/day	4,901 (2,464)	5,659 (2,611)	-758 (-1,037 to -479)
Hemoglobin A1C^a^, %	7.6 (1.3)	7.7 (1.3)	-0.10 (-0.21 to 0)
Systolic blood pressure, mm Hg	137 (16)	133 (14)	4.0 (2.3 to 5.7)
Diastolic blood pressure, mm Hg	80 (10)	79 (9)	1.4 (0.4 to 2.4)
BMI, kg/m^2^	30.2 (6.0)	30.2 (6.0)	0.07 (-0.04 to 0.18)
Waist circumference, cm			
Women	98.7 (14.1)	98.9 (13.8)	-0.2 (-1.0 to 0.5)
Men	104.2 (13.0)	104.8 (12.7)	-0.5 (-1.3 to 0.2)
Waist/hip			
Women	0.879 (0.063)	0.882 (0.061)	-0.003 (-0.01 to 0.004)
Men	0.967 (0.060)	0.971 (0.060)	-0.004 (-0.01 to 0.004)
Self-reported measures			
Total energy intake, kcal/day^b^	1,730 (684)	1,700 (662)	30.0 (-37.9 to 98.0)
Carbohydrate intake, g/day^b^	187 (76)	182 (74)	5.7 (-2.9 to 14.4)
Salt intake, mg/day^b^	2,598 (1353)	2,546 (1182)	51.9 (-86.3 to 190)
Total physical activity, metabolic equivalent-minutes/week^c^	2,598 (2463)	2,921 (2700)	-451 (-872 to -30.4)

SD, standard deviation; CI, Confidence interval

^a ^Given that A1C reflects glycemic control over the prior two to three months, winter/spring A1C values may reflect fall/winter glycemic control and summer/fall A1C values may reflect spring/summer glycemic control. We therefore also computed Winter/spring to Summer/Fall difference in A1C which was 0.012% (95% CI -0.10% to 0.13%).

^b ^Shatenstein and colleagues' Food Frequency Questionnaire [21]

^c ^International Physical Activity Questionnaire (Short Last 7 days format) [20]

The fall/winter -spring/summer daily step difference was similar in women (-734 steps/day, 95% CI -1,079 to -388) and men (-781 steps/day, 95% CI -1,222 to -341). The fall/winter - spring/summer self-reported total physical activity difference was also similar in women and men but confidence intervals were wide in sex-specific analyses. The difference in systolic blood pressure was similar in women (4.5 mm Hg, 95% CI 2.1 to 6.8 mm Hg) and men (3.5 mm Hg, 95% CI: 0.9 to 6.1). We could not, however, conclusively establish that the fall/winter increase in systolic blood pressure detected was attributable to the fall/winter reduction in daily steps.

When analyses were restricted to the 118 individuals who did not experience a change in the number of antihypertensive medications between winter and summer, the overall winter-summer difference in systolic blood pressure remained (4.1 mm Hg, 95%: CI 1.3 to 6.9).

## Discussion

Through a longitudinal cohort study, we have demonstrated a 15% reduction in daily steps, a 4.0 mm Hg increase in systolic blood pressure, and a 1.4 mm Hg increase in diastolic blood pressure during the fall and winter months among overweight adults treated for type 2 diabetes. However, no clinically important seasonal variations in A1C were identified. The magnitude of reduction in daily steps in fall and winter was not sufficient to explain the corresponding fall/winter increase in blood pressure, suggesting that other seasonal factors impact blood pressure. Nonetheless, both the 15% reduction in daily steps and the blood pressure increase may arguably have a long term impact on vascular health, particularly as these may recur annually.

With respect to daily step counts, our participants in fact demonstrated low daily step counts throughout the year. Specifically, average daily step counts were 5,659 during the spring/summer period, decreasing to 4,901during the fall/winter period, with a mean fall/winter to spring/summer within-individual difference of -758 (95% CI: -1,037 to -479). The 758 steps/day reduction that we have identified is consistent with data from nondiabetic samples, including a 900 steps/day reduction in an American study [22] and a 1,300 steps/day reduction in a British study [23]. A daily step reduction in already sedentary diabetes patients is arguably of particular significance given the high risk for vascular complications in this clinical population [1-3] combined with the survival benefits of higher activity levels[4,5].

With respect to A1C levels, the between-season and between-period differences that we identified were in the order of 0.1% with wide confidence intervals: our study was powered to detect differences of 0.2% or more. While we cannot conclusively exclude the existence of a between-season difference, such a 0.1% difference would arguably be of questionable clinical importance. Our finding of relatively stable A1C levels contrasts with some previous reports [9,12,13]. Unlike these previous studies, we assessed average differences *within *individuals rather than across individuals; such within-individual differences may be less marked. Further, stability of A1C levels may also have resulted from the fact that this was a treated clinical population specifically managed for type 2 diabetes and thus subject to careful titration of antihyperglycemic medication. Moreover, given that these individuals volunteered to participate in an observational cohort study, they may have been somewhat more diligent in their diabetes self-management than other diabetes patients.

In contrast, we did detect a fall/winter systolic blood pressure increase, despite the possibility of more careful self-management. Fall/winter was associated with an increase in systolic blood pressure levels of 4.0 mm Hg (95% CI: 2.3 to 5.7) and an increase in diastolic blood pressure of 1.4 (0.4 to 2.4). In other populations, fall/winter increases in systolic blood pressure have been reported at roughly 2 to over 5 mm Hg [15,16]. The increase that we have detected is consistent with these values. The importance, however, of such a blood pressure increase is arguably greater in adults with diabetes. The United Kingdom Prospective Diabetes Study (UKPDS) [24], and more recently, the Action in Diabetes and Vascular Disease (ADVANCE) trial [25], have demonstrated the particular importance of lower blood pressure levels in patients with type 2 diabetes. In the UKPDS, each 10 mm Hg reduction in systolic blood pressure was associated with a 15% lower mortality and an 11% lower heart attack rate. The systolic blood pressure lowering that we have detected in spring/summer represents approximately one third of such a 10 mm Hg reduction. In ADVANCE, treatment with perindopril/indapamide was associated with a mean systolic blood pressure reduction of 5.6 mm Hg and a 9% reduction in CVD events. The systolic blood pressure lowering that we have detected in spring/summer is therefore equivalent to more than half of the systolic blood pressure reduction in the ADVANCE trial. Preventing even a small recurrent annual blood pressure increase in fall/winter may be of clinical significance as it could potentially result in cumulative vascular damage over time.

The large fall/winter-spring/summer difference in blood pressure that we detected could not be explained by the magnitude of the fall/winter-spring/summer daily step difference that we observed. Similarly, corresponding differences in anthropometric parameters and salt intake (Table 2) were not sufficient to account for the blood pressure difference. Alternate potential contributory factors may be a direct impact of cold temperatures on blood pressure [26,27], lower sunlight exposure and reduced vitamin D production in fall/winter [28], or acute illnesses such as influenza.

The average 758 daily step reduction did not lead to seasonal increases in A1C in our cohort and did not account for the mean 4.0 mm Hg systolic blood pressure increase in fall and winter. This should not, however, deter clinicians from encouraging their patients to increase their step counts as a means of lowering both A1C and blood pressure. In one interventional study conducted among type 2 diabetes patients, for example, an increase in daily steps of more than 2,300 steps/day led to a 0.3% reduction in A1C [29]. Furthermore, in a previously-reported analysis of our cohort aiming to cross-sectionally assess associations between habitual daily step counts and systolic blood pressure, we identified an inverse relationship between daily steps and blood pressure among women treated for diabetes [30]. Specifically, a 1,000 daily step increment among women was conclusively related to a -2.6 mm Hg change in systolic and a -1.4 mm Hg change in diastolic blood pressure. Among men, changes were signalled but were smaller and inconclusive (-0.7 and -0.6 mm Hg, respectively). Given the potential for impact of higher daily steps on A1C, blood pressure, and long term mortality [4,5], the low habitual daily step counts and fall/winter reduction that we have quantified in the present paper signal a need for action

Targeting daily step counts in type 2 diabetes has been the focus of a previous Canadian trial, Tudor-Locke's First Step program [31]. In this trial, group meetings and pedometer-based self-monitoring was successful in achieving a 3,000 daily step increase at 16 weeks but between-group differences were not sustained at longer term follow-up following the intervention period. There thus appears to be a need for ongoing monitoring and encouragement, ideally through a strategy that is better integrated into routine clinical care. Our own discussions with adults with type 2 diabetes indicate a need for such long term support [32]. In this regard, the physician-patient relationship is a logical potential source of such support, given the continuing association. Follow-up of pedometer-based self-monitoring records would be highly compatible with existing systems of diabetes care, wherein, for example, patient records of capillary glucose monitoring and home blood pressure measurements are often reviewed and accounted for in medication titration. Our quantification of daily steps and fall/winter reductions provides a framework for discussion and monitoring.

We acknowledge some limitations. The repeated visits in the context of an observational cohort study required particularly dedicated volunteers who may not be representative of all diabetes patients. However, we would argue that other patients are thus likely to have even lower daily step counts and greater fall/winter step count reductions and blood pressure increases. A second potential limitation is that blood pressure values were only assessed once per visit and thus the overall levels reported may be higher than if serial measurements had been averaged. However, a standard procedure was followed and thus between visit differences were likely accurate. Strengths of our study include the examination of within-individual comparisons, precluding the need for further adjustments.

## Conclusions

Our observational cohort study is the largest longitudinal study to assess daily step counts in type 2 diabetes. Step counts averaged at the sedentary level in fall/winter (mean 4,901 steps/day, SD 2,464) and at the low active level in spring/summer (mean 5,659 steps/day, SD 2,611). There was a -758 (95% CI: -1,037 to -479) mean fall/winter to spring/summer within-individual difference. Although no important seasonal difference in A1C was detected, there was a 4.0 mm Hg systolic blood pressure increase and a 1.4 mm Hg diastolic blood pressure increase during the fall/winter period. This information, in conjunction with the existing body of evidence supporting pedometer-based strategies, may be used by clinicians in setting step count targets with their patients and preventing both fall/winter declines in step counts and increases in blood pressure. For these reasons, our findings bring to light an opportunity for a pre-emptive approach to vascular risk reduction in type 2 diabetes.

## Competing interests

The authors declare that they have no competing interests.

## Authors' contributions

KD led the design of the present study and obtained the necessary funding; she supervised all data collection procedures and analyses; wrote the original draft and made revisions based on co-authors' comments. LJ (biostatistician) provided sample size estimates, developed the analytic plan, and co-supervised all statistical analyses. All authors made important contributions at the study design phase, provided critical comments during manuscript preparation, read and approved the final manuscript.

## References

[B1] Booth GL Kapral MK Fung K Tu JV Relation between age and cardiovascular disease in men and women with diabetes compared with non-diabetic people: a population-based retrospective cohort study Lancet 2006 368 29 36 10.1016/S0140-6736(06)68967-8 16815377

[B2] Lee WL Cheung AM Cape D Zinman B Impact of diabetes on coronary artery disease in women and men: a meta-analysis of prospective studies Diabetes Care 2000 23 962 968 10.2337/diacare.23.7.962 10895847

[B3] Gu K Cowie CC Harris MI Mortality in adults with and without diabetes in a national cohort of the U.S. population, 1971-1993 Diabetes Care 1998 21 1138 1145 10.2337/diacare.21.7.1138 9653609

[B4] Gregg EW Gerzoff RB Caspersen CJ Williamson DF Narayan KM Relationship of walking to mortality among US adults with diabetes Arch Intern Med 2003 163 1440 1447 10.1001/archinte.163.12.1440 12824093

[B5] Hu F Stampfer M Solomon C Liu S Colditz G Speizer F Willett W Manson J Physical activity and risk for cardiovascular events in diabetic women Ann Intern Med 2001 134 96 105 11177312 10.7326/0003-4819-134-2-200101160-00009

[B6] Ford E Herman W Leisure-time physical activity patterns in the U.S. diabetic population. Findings from the 1990 National Health Interview Survey--Health Promotion and Disease Prevention Supplement Diabetes Care 1995 18 27 33 10.2337/diacare.18.1.27 7698044

[B7] Wing R Goldstein M Acton K Birch L Jakicic J Sallis J Jr Smith-West D Jeffery R Surwit R Behavioral science research in diabetes: lifestyle changes related to obesity, eating behavior, and physical activity Diabetes Care 2001 24 117 123 10.2337/diacare.24.1.117 11194216

[B8] Ishii H Suzuki H Baba T Nakamura K Watanabe T Seasonal variation of glycemic control in type 2 diabetic patients. Letter Diabetes Care 2001 24 1503 10.2337/diacare.24.8.1503 11473100

[B9] Sohmiya M Kanazawa I Kato Y Seasonal changes in body composition and blood HbA1c levels without weight change in male patients with type 2 diabetes treated with insulin Diabetes Care 2004 27 1238 1239 10.2337/diacare.27.5.1238 15111560

[B10] Chen H Jap T Chen R Lin H A prospective study of glycemic control during holiday time in type 2 diabetic patients Diabetes Care 2004 27 326 330 10.2337/diacare.27.2.326 14747208

[B11] Asplund J Seasonal variation of HbA1c in adult diabetic patients. Letter Diabetes Care 1997 20 234 9118784 10.2337/diacare.20.2.234a

[B12] Carney T Guy S Helliwell C Seasonal variation in HbA1c in patients with Type 2 diabetes mellitus. Letter Diabetic Med 2000 17 554 555 10.1046/j.1464-5491.2000.00311-5.x 10972592

[B13] Tseng C Brimacombe M Xie M Rajan M Wang H Kolassa J Crystal S Chen T Pogach L Safford M Seasonal patterns in monthly hemoglobin A1c values Am J Epidemiol 2005 161 565 574 10.1093/aje/kwi071 15746473

[B14] Barnett AG Sans S Salomaa V Kuulasmaa K Dobson AJ The effect of temperature on systolic blood pressure Blood Press Monit 2007 12 195 203 10.1097/MBP.0b013e3280b083f4 17496471

[B15] Ulmer H Kelleher C Diem G Concin H Ruttmann E Estimation of seasonal variations in risk factor profiles and mortality from coronary heart disease Wien Klin Wochenschr 2004 116 662 668 10.1007/s00508-004-0222-x 15941075

[B16] Alperovitch A Lacombe JM Hanon O Dartigues JF Ritchie K Ducimetiere P Tzourio C Relationship between blood pressure and outdoor temperature in a large sample of elderly individuals: the Three-City study Arch Intern Med 2009 169 75 80 10.1001/archinternmed.2008.512 19139327

[B17] Dasgupta K Chan C Da Costa D Pilote L De Civita M Ross N Strachan I Sigal R Joseph L Walking behaviour and glycemic control in type 2 diabetes: seasonal and gender differences - Study design and methods Cardiovasc Diabetol 2007 15 6 1 10.1186/1475-2840-6-1 PMC1783642 17224062

[B18] Cyarto E Myers A Tudor-Locke C Pedometer accuracy in nursing home and community-dwelling older adults Med Sci Sports Exerc 2005 36 205 209 10.1249/01.MSS.0000113476.62469.98 14767241

[B19] Tudor-Locke C Bassett DR Jr How many steps/day are enough? Preliminary pedometer indices for public health Sports Med 2004 34 1 8 10.2165/00007256-200434010-00001 14715035

[B20] Scoring protocol http://www.ipaq.ki.se/scoring.htm

[B21] Shatenstein B Nadon S Godin C Ferland G Development and validation of a food frequency questionnaire Can J Diet Pract Res 2005 66 67 75 10.3148/66.2.2005.67 15975195

[B22] Tudor-Locke C Bassett D Swartz A Strath S Parr B Reis J Dubose K Ainsworth B A preliminary study of one year of pedometer self-monitoring Ann Behav Med 2004 28 158 162 10.1207/s15324796abm2803_3 15576253

[B23] Hamilton SL Clemes SA Griffiths PL UK adults exhibit higher step counts in summer compared to winter months Ann Hum Biol 2008 35 154 169 10.1080/03014460801908058 18428010

[B24] Tight blood pressure control and risk of macrovascular and microvascular complications in type 2 diabetes: UKPDS 38. UK Prospective Diabetes Study Group BMJ 1998 317 703 713 9732337 PMC28659

[B25] Patel A MacMahon S Chalmers J Neal B Woodward M Billot L Harrap S Poulter N Marre M Cooper M Effects of a fixed combination of perindopril and indapamide on macrovascular and microvascular outcomes in patients with type 2 diabetes mellitus (the ADVANCE trial): a randomised controlled trial Lancet 2007 370 829 840 10.1016/S0140-6736(07)61303-8 17765963

[B26] Liang WW Seasonal changes in preprandial glucose, A1C, and blood pressure in diabetic patients Diabetes Care 2007 30 2501 2502 10.2337/dc07-0597 17586743

[B27] Modesti PA Morabito M Bertolozzi I Massetti L Panci G Lumachi C Giglio A Bilo G Caldara G Lonati L Weather-related changes in 24-hour blood pressure profile: effects of age and implications for hypertension management Hypertension 2006 47 155 161 10.1161/01.HYP.0000199192.17126.d4 16380524

[B28] Wang TJ Pencina MJ Booth SL Jacques PF Ingelsson E Lanier K Benjamin EJ D'Agostino RB Wolf M Vasan RS Vitamin D deficiency and risk of cardiovascular disease Circulation 2008 117 503 511 10.1161/CIRCULATIONAHA.107.706127 18180395 PMC2726624

[B29] Kempf K Kruse J Martin S ROSSO-in-praxi: a self-monitoring of blood glucose-structured 12-week lifestyle intervention significantly improves glucometabolic control of patients with type 2 diabetes mellitus Diabetes Technol Ther 2010 12 547 553 10.1089/dia.2010.0008 20597830

[B30] Manjoo P Joseph L Pilote L Dasgupta K Sex Differences in Step Count-Blood Pressure Association: A Preliminary Study in Type 2 Diabetes PLoS One 2010 5 e14086 10.1371/journal.pone.0014086 21124929 PMC2989914

[B31] Tudor-Locke C Bell R Myers A Harris S Ecclestone N Lauzon N Rodger N Controlled outcome evaluation of the First Step Program: a daily physical activity intervention for individuals with type II diabetes Int J Obes Relat Metab Disord 2004 28 113 119 10.1038/sj.ijo.0802485 14569279

[B32] Casey D De Civita M Dasgupta K Understanding physical activity facilitators and barriers during and following a supervised exercise programme in Type 2 diabetes: a qualitative study Diabetic Med 2010 27 79 84 10.1111/j.1464-5491.2009.02873.x 20121893

